# Linking IFN-γ-Mediated Pathogenesis to ROCK-Targeted Therapy in a Scalable iPSCs-Based Vitiligo Model

**DOI:** 10.3390/ijms26168069

**Published:** 2025-08-21

**Authors:** Toshiro Komatsu, Yupeng Dong, Takaharu Ikeda, Tamihiro Kawakami

**Affiliations:** 1 Division of Dermatology, Tohoku Medical and Pharmaceutical University, Sendai 981-8558, Miyagi, Japan; komatsu-hifuka@khc.biglobe.ne.jp (T.K.); t-ikeda@tohoku-mpu.ac.jp (T.I.); vasculitisdermatology@hotmail.com (T.K.); 2 Kawakami Dermatology Clinic, Matsudo 270-0034, Chiba, Japan

**Keywords:** iPS, melanocyte, keratinocyte, co-culture, ROCK inhibitor, IFN-γ, Vitiligo, bFGF, ET-1, dendrites

## Abstract

Vitiligo is a chronic autoimmune dermatosis defined by selective melanocyte depletion and patchy depigmentation. IFN–γ-driven recruitment of autoreactive CD8^+^ T cells and induction of melanocyte apoptosis are central to its pathogenesis. Current therapies—including UVB phototherapy, tacrolimus, vitamin D3 analogs, and surgical methods—show limited and inconsistent efficacy. Emerging treatments like JAK inhibitors and WNT activators offer potential but require further validation. Translational progress is hindered by a lack of scalable human models. Here, we describe a tunable in vitro vitiligo platform in which human iPSC-derived melanocytes (iMc) are co-cultured with keratinocytes on Matrigel and exposed to precise graded IFN-γ concentrations. Our data revealed dose-dependent decreases in iMc survival and dendritic structure, faithfully mirroring derived melanocyte pathology. Leveraging this platform, we first evaluated the short-term efficacy of the ROCK inhibitor Y27632 under early-stage patient IFN-γ concentrations representative of patient lesional thresholds. At three days, Y27632 significantly upregulated adhesion molecules E-cadherin and DDR1, and two central factors—ET1 and bFGF. Importantly, ROCK inhibition reversed dendritic retraction and improved overall viability of iMc-keratinocytes. These findings position ROCK blockade as a promising adjunctive strategy and establish a pre-clinical platform for evaluating combination therapies for durable pigment restoration.

## 1. Introduction

Vitiligo is a chronic autoimmune dermatosis characterized by selective loss of epidermal melanocytes and the emergence of depigmented macules [1,2], affecting approximately 1% of the global population and imposing substantial psychosocial morbidity [3,4,5]. Its multifactorial pathogenesis encompasses oxidative stress, neuroendocrine dysregulation [6,7], and, centrally, interferon γ (IFN-γ)–driven JAK–STAT1 signaling [8,9,10,11,12,13], which orchestrates CXCL9/CXCL10-mediated CXCR3^+^ CD8^+^ T cell recruitment and activates p21-dependent melanocyte apoptosis, potentiating oxidative damage and cellular senescence [14,15,16,17,18,19]. Such WNT/β-catenin signaling attenuation also causes melanocyte differentiation defects and survival impairment, leading to depigmentation [20,21,22]. IFN-γ can suppress WNT/β-catenin–Melanocyte inducing transcription factor (MITF) signaling and precipitate melanocyte senescence and apoptosis through upregulating inhibitory factors (e.g., DKK1, sFRP2) in keratinocytes [22,23,24].

Although primary melanocytes from vitiligo lesions most faithfully reflect disease phenotypes, their limited proliferative capacity [25,26] and genetic instability constrain experimental use [5,27], and direct reprogramming of patient-derived melanocytes into iPSCs remains unestablished, forcing reliance on melanocytes from normal skin or fibroblast- or blood-derived lines [28,29,30,31]. Likewise, full thickness 3D skin equivalents recapitulate epidermal architecture mainly formed by keratinocytes, fibroblast and collagen [32], but lack melanocytes and key immune effectors such as CXCR3^+^ T cells and macrophages [13,18,33,34,35]. Currently, human full-thickness skin surgical explants cultured in vitro can maintain skin architecture and the native microenvironment—including vasculature and neural components—with preserved function over several days [29,30]. This model holds great translational medicine potential; however, its preclinical translational value requires reproducible mechanistic studies via collaborative efforts. Consequently, there is an urgent need for a stable, easily expandable in vitro platform that combines physiological relevance with experimental tractability to bridge the gap between basic medical research, preclinical drug screening and clinical translation. To address this need, we first established a novel method to induce iPSC to antibiotic- and serum-free, easy-to-handle, high-quality melanocytes (iMc) [28,36,37], which revealed that kojic acid decreased eumelanin. As we have reported that under vitiligo patient primary melanocytes and human normal keratinocytes co-culture conditions [25], the Rho-associated kinase ROCK inhibitor increased the expression of melanocyte survival and adherence pathways [38,39,40,41,42]—basic fibroblast growth factor (bFGF) [43,44,45,46,47,48,49] and E-cadherin [50,51,52], discoidin domain receptor tyrosine kinase 1 (DDR1) [52,53] and Glycoprotein NMB (GPNMB) [54]—which are essential for maintaining dendritic integrity, cell adherence and promoting proliferation via MAPK/p38, MITF-GPNMB expression [54,55,56,57,58]. On the other hand, we have developed a Matrigel-based co-culture of iMc and primary keratinocytes [26,37]. Here, we introduce that this iMc-keratinocyte co-culture system is easy to expand and permits precise, dose-dependent IFN-γ challenge and sensitive quantification of early melanocyte pathology which is confirmed by comparing vitiligo patient-derived primary melanocytes with iMc, both co-cultured with human normal keratinocytes. Building on the use of ROCK inhibitor in our previous studies, we first used this vitiligo model platform to evaluate whether ROCK inhibition can restore melanocyte–keratinocyte crosstalk and counteract IFN-γ–driven dysfunction, thereby offering a novel avenue for therapeutic repigmentation.

## 2. Results

### 2.1. Comparing Two Co-Culture Systems: iMc and Primary Vitiligo Melanocytes Co-Culture System

#### 2.1.1. iMc Co-Culture with Keratinocytes System

**Figure 1:**
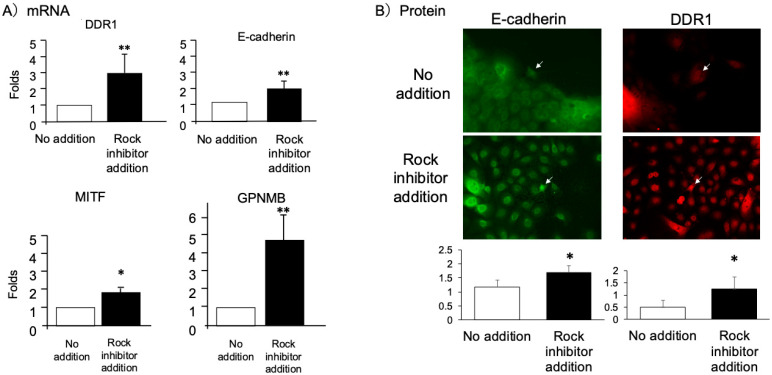
Y27632 Upregulates Gene Expression in the iMc–Keratinocyte Co-Culture System. (**A**) Human iPSC-derived melanocytes (iMc) and primary keratinocytes were co-cultured in the presence or absence of the ROCK inhibitor Y27632 (10 μM) for 3 days. mRNA expression levels of adhesion molecules (E-cadherin, DDR1), and in addition, melanocyte-specific genes (MITF (TF), GPNMB; analyzed in iMc monoculture) were quantified by RT-qPCR. (**B**) Protein expression levels of E-cadherin, DDR1 were evaluated under the same culture conditions. White arrows indicate iMc. Data are presented as mean ± SEM from independent experiments. Statistical significance was determined by unpaired two-tailed *t*-test: * *p* < 0.05; ** *p* < 0.001; N.S., not significant.

Consistent with observations in primary melanocytes described in introduction, treatment with Y-27632 significantly enhanced expression of melanocyte adhesion markers E-cadherin and DDR1 (Figure 1). Moreover, Y-27632 also upregulated MITF and its downstream effector GPNMB, both essential for melanogenesis and dendritic maintenance (Figure 1A). While Stem Cell Factor (SCF) exhibited variable expression, the ROCK inhibitor clearly enhanced key survival, adhesion and pigmentation Markers in our iMc co-culture system.

#### 2.1.2. Vitiligo Melanocytes Co-Culture Keratinocytes

**Figure 2:**
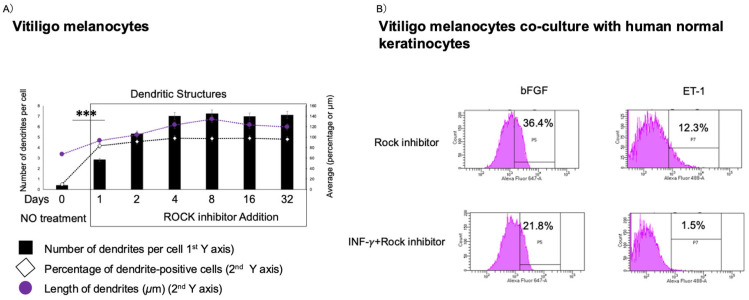
ROCK Inhibition Restores Dendritic Integrity and Trophic Factor Expression in a Vitiligo primary Melanocyte Co-Culture Model. (**A**) Long-term culture of vitiligo patient-derived primary melanocytes demonstrated that treatment with the ROCK inhibitor Y27632 (10 μM) rapidly restored dendritic arborization, as quantified using the Melanocyte Dendrite Imaging Score (MDIS) method. (**B**) In the vitiligo patient derived melanocyte-keratinocyte co-culture system, pretreatment with Y27632 (10 μM) for 3 days attenuated the IFN-γ (10 ng/mL)–induced suppression of basic fibroblast growth factor (bFGF) and endothelin-1 (ET-1), at the protein level. Data are expressed as mean ± SEM. Statistical significance was determined using unpaired two-tailed *t*-tests; *** *p* < 0.0001.

In prior work, we demonstrated that the ROCK inhibitor Y-27632 could upregulate bFGF expression in co-cultures of primary melanocytes derived from vitiligo patients and keratinocytes. In this study, ROCK inhibition also visibly restored dendritic architecture of vitiligo melanocytes within 24 h, quantified using a novel imaging-based scoring system, detail see materials and Methods, the Melanocyte Dendritic Imaging Score (MDIS) (Figure 2A).

Taken together, ROCK inhibitor broadly upregulated similar expression of genes in both co-culture systems.

### 2.2. Expand to Establish an IFN-γ–Induced Vitiligo Model

#### 2.2.1. IFN-γ in Vitiligo Melanocytes Co-Culture Keratinocytes

After adding IFN-γ (10 ng/mL) into medium [materials and methods, [11,12], vitiligo co-cultured cells showed significant reductions in protein levels of basic fibroblast growth factor (bFGF) (36.4% to 21.8%) and endothelin-1 (ET-1) (12.3% to 1.5%) (Figure 2B). Notably, pre-treatment with Y-27632 partially failed to block this IFN-γ-induced suppression.

#### 2.2.2. IFN-γ–Induced Skin Injury in a Three-Dimensional Skin Model (T-Skin™)

**Figure 3:**
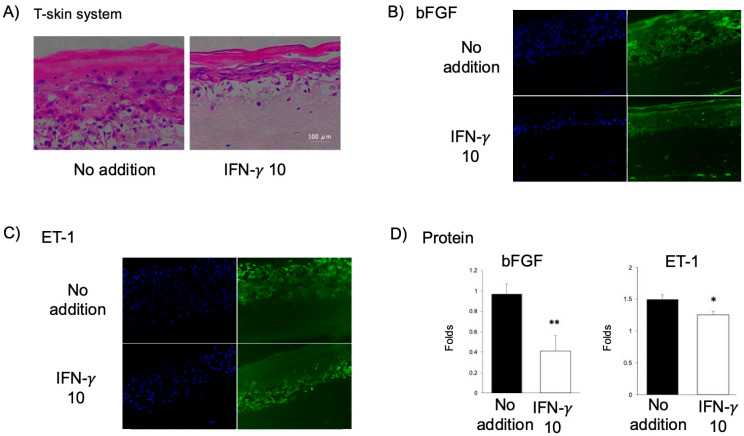
Y27632 Rescues bFGF and ET-1 in a 3D Skin Model. (**A**) Hematoxylin and eosin (H&E) staining of reconstructed human skin (T-Skin^TM^) shows histological changes following treatment with or without IFN-γ (10 ng/mL) for 3 days. (**B**–**D**) Under the same conditions, protein expression levels were evaluated. Immunofluorescence staining and subsequent quantitative analysis using ImageJ 1.53 revealed that Y27632 treatment restored the expression of basic fibroblast growth factor (bFGF, **B**) and endothelin-1 (ET-1, **C**), with densitometric quantification shown in (**D**). Data are presented as mean ± SEM. Statistical significance: * *p* < 0.05; ** *p* < 0.001.

To model the pathological progress by IFN-γ triggered cell death in human skin, a three-dimensional full-thickness skin model (T-Skin™) constructed by keratinocytes, fibroblasts, and collagen was used as a skin control. Although obtaining consistent data across different skin tissue samples proved challenging, HE stains revealed that administration of 10 ng/mL IFN-γ led to marked histological alterations including epidermal thinning, reduced extracellular matrix deposition, and partial loss of both fibroblasts and keratinocytes (Figure 3A). Immunofluorescence staining revealed significant downregulation of two key factors we used as cell dysfunction markers, basic fibroblast growth factor (bFGF) and endothelin-1 (ET-1), both predominantly expressed by keratinocytes (Figure 3B–D).

#### 2.2.3. Dose-Dependent IFN-γ Cytotoxicity in iMC Co-Cultures System

**Figure 4:**
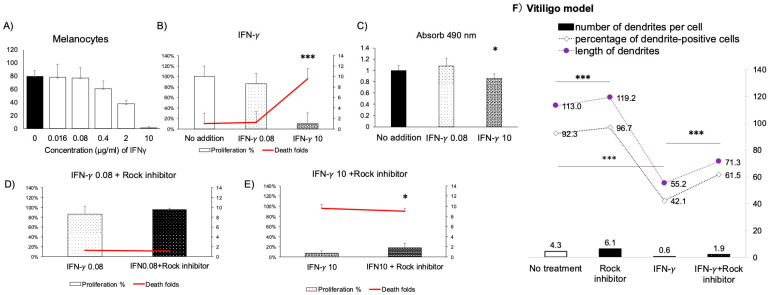
ROCK Inhibitor Rescues IFN-γ–Induced Dysfunction in iMc (**A**) iMc were treated with increasing concentrations of IFN-γ, and cell viability was assessed using the MTS assay. (**B**,**C**) In the iMc–keratinocyte co-culture system, IFN-γ induced melanocyte death and inhibited proliferation (**B**) as well as melanin synthesis (**C**). (**D**,**E**) Co-administration of the ROCK inhibitor Y27632 restored cell viability and proliferation under low (0.08 ng/mL, **D**) and high (10 ng/mL, **E**) IFN-γ conditions. (**F**) ROCK inhibition also rescued dendritic structure loss induced by 0.08 ng/mL IFN-γ, as quantified using the melanocyte dendritic imaging score (MDIS) method. * *p* < 0.05; *** *p* < 0.0001. Comparisons were made between indicated two groups or against the untreated control group as specified.

To model progressive stages of vitiligo, we examined IFN-γ dose responses in iMc–keratinocyte co-cultures. Under early-stage patient IFN-γ concentrations representative of patient lesional thresholds, low-dose IFN-γ (0.08 ng/mL = 80 pg/mL), approximating early-stage inflammatory conditions, did not affect cell viability significantly, whereas high-dose 10 ng/mL induced substantial melanocyte apoptosis and reduced melanin content (Figure 4A–C) mimics vitiligo lesions.

### 2.3. ROCK Inhibitor Partially Rescues IFN-γ–Induced Trophic Factor Suppression

Based on our investigations in both iMc–keratinocyte and vitiligo patient–derived melanocyte-keratinocyte co-culture systems, ROCK inhibition represents the primary therapeutic strategy evaluated in this study. Y-27632 significantly mitigated IFN-γ–induced cytotoxicity, as evidenced by improved cell viability (Figure 4E). Notably, MDIS analysis showed that dendritic complexity dropped to 42.1% under IFN-γ (0.08 ng/mL) alone for 3 days but recovered to 61.5% with Y-27632 co-treatment, even exceeding control levels in some metrics (92.3% vs. 96.7%) (Figure 4F).

**Figure 5:**
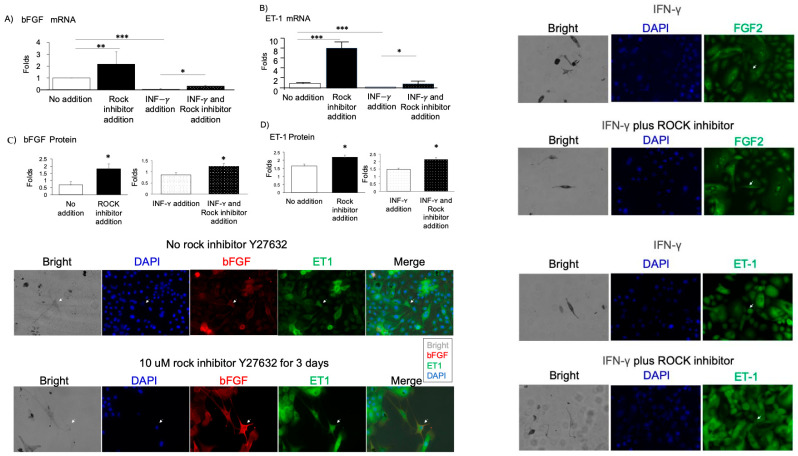
ROCK Inhibitor Restores IFN-γ–Suppressed bFGF and ET-1 Expression in the iMc–Keratinocyte Co-Culture System. In the iMc and primary keratinocyte co-culture system, the effects of IFN-γ (10 ng/mL) and/or ROCK inhibitor Y27632 (10 μM) on melanocyte survival factors were assessed. (**A**,**B**) RT-qPCR analysis revealed changes in mRNA expression of basic fibroblast growth factor (bFGF) and endothelin-1 (ET-1). (**C**,**D**) Immunofluorescence staining followed by ImageJ quantification showed corresponding protein level changes in bFGF (**C**) and ET-1 (**D**). White arrows indicate iMc. Statistical significance is indicated as follows: * *p* < 0.05, ** *p* < 0.001, *** *p* < 0.0001. Comparisons were made between the indicated groups or against the untreated control group.

Building on these findings, we directly tested whether ROCK inhibition could reverse IFN-γ–mediated suppression of bFGF and ET-1 in the iMc–keratinocyte co-culture system. RT-qPCR and immunofluorescence staining revealed that both mRNA and protein levels of bFGF and ET-1 were significantly downregulated upon IFN-γ (80 pg/mL) treatment (Figure 5A,B). However, Y-27632 supplementation markedly restored expression levels of both factors, consistent with observations in primary melanocyte cultures. Quantitative image analysis confirmed this rescue effect, with partial but statistically significant recovery of bFGF and ET-1 expression in the presence of both IFN-γ and the ROCK inhibitor (Figure 5C,D).

## 3. Discussion

**Figure 6:**
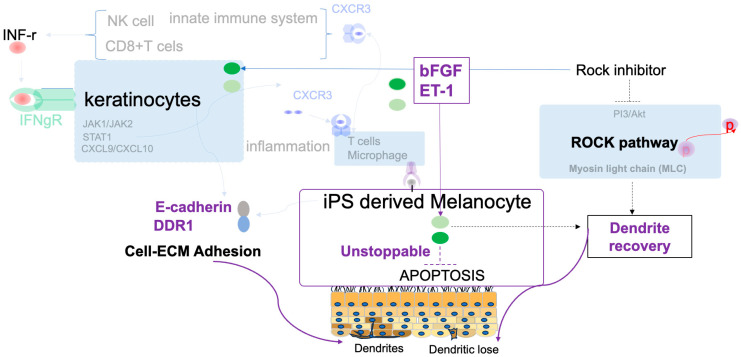
Schematic Overview of ROCK Inhibition in Vitiligo Pathogenesis Using an IFN-γ–Induced Co-Culture of iMc and Keratinocytes. The current understanding of vitiligo pathogenesis involves IFN-γ signaling inducing CXCL9/10 expression from keratinocytes via the JAK–STAT1 axis, leading to autoreactive CD8^+^ T cell recruitment and melanocyte apoptosis. In this study, we established a reproducible in vitro platform modeling the early pathogenic phase of vitiligo by co-culturing iMc with human keratinocytes under IFN-γ stimulation. Using this system, we demonstrated that although ROCK inhibition (via Y27632) does not fully prevent IFN-γ–induced melanocyte damage, it partially restores critical morphological and functional features—such as dendritic complexity, cell survival, and expression of key factors including bFGF, ET-1, E-cadherin, DDR1.

In this study, we developed and validated a physiologically relevant co-culture model using iMc and primary keratinocytes that recapitulates early IFN-γ–driven melanocyte injury [6,9,10,11,12,13,18]. Through this model, we show that ROCK inhibition by Y-27632 confers multifaceted protection against IFN-γ–induced dysfunction by most importantly recovering the dendrite-like structure of iMc even particularly as shown in Figure 4F. Remarkably, dendritic complexity was preserved or even enhanced relative to controls, highlighting the rapid and functional impact of ROCK inhibition. These results were consistent across both patient-derived and iPSC-derived model, underscoring the translational relevance of this approach. Mechanistically (Figure 6), Y-27632 enhances the expression of E-cadherin and DDR1 (Figure 1), which stabilizes adherens junctions and preserves melanocyte residency within the basal epidermis; these adhesion molecules facilitate melanosome transfer and intercellular communication with keratinocytes—crucial processes disrupted in early vitiligo [51,52,53,59,60,61]. Importantly, under iMC co-culture conditions, Y-27632 also upregulated MITF and its downstream effector GPNMB (Figure 1), both critical for dendritic integrity, pigment synthesis, and cell survival [54,62,63,64].

Beyond adhesion (Figure 6), Y-27632 selectively restored bFGF and ET-1(Figure 5), two trophic factors primarily secreted by keratinocytes that support melanocyte proliferation, migration, and melanogenesis through the MAPK–MITF axes [52,54,57,58]. On the other hand, in the Y-27632 pre-treatment condition, IFN-γ markedly suppressed ET-1 levels (from 12.5% to 1.5% in Figure 2B), indicating a pronounced downregulation of ET-1—likely most in keratinocytes given the 1:10 melanocyte-to-keratinocyte seeding ratio—which is associated with dendrite loss [65,66]. Moreover, in the iMc co-culture model, IFN-γ markedly suppressed bFGF and ET-1 expression; however, co-treatment with a ROCK inhibitor partially restored their expression at both the transcriptional and protein levels (Figure 5). This restoration may reflect ROCK’s modulatory role on downstream or parallel nodes of the JAK–STAT pathway, especially at the level of STAT1/STAT3 phosphorylation [67,68], also suggests involvement of additional regulatory networks. Both WNT/β-catenin [19,24,69], and NF-κB pathways [58,70] have roles in melanocyte survival and repigmentation responses and crosstalk between ROCK and these axes may underpin synergistic effects. We suggest that combined ROCK and WNT pathway activation could yield additive benefits while maintaining tighter control over proliferative cues to minimize oncogenic risk. Although this study tested ROCK inhibition alone, our iMc–keratinocyte platform is well-suited to evaluate combination strategies. We therefore anticipate that Y-27632 combined with agents targeting JAK, WNT, or MAPK could suppress IFN-γ-driven inflammation while enhancing pro-survival and melanogenic signaling; systematic testing of such combinations is a clear direction for future work.

From a platform perspective, our iMc co-culture model offers a scalable, tunable system for interrogating early-stage melanocyte dysfunction in vitiligo. However, it lacks critical immune effectors such as CXCR3^+^ CD8^+^ T cells and macrophages, which drive tissue remodeling and chronic inflammation [14,16,18], and we have not demonstrated keratinocyte expression of CXCL3/CXCL9/CXCL10 in this system. Future integration and expanding with patient-derived immune cells or adaptation to 3D skin organoids will enable deeper insights into direct immune–melanocyte interactions and validate ROCK inhibitor efficacy under conditions mimicking in vivo autoimmunity [29,30].

Despite long-term safety concerns, ROCK inhibitors have been widely used in clinical settings [71,72]. While our data support short-term benefits in restoring melanocyte structure and function [73,74,75], rigorous long-term studies—especially in models predisposed to oncogenesis—are necessary to rule out potential neoplastic transformation.

Clinically, ROCK inhibition or targeting bFGF emerges as a compelling adjunct to current vitiligo therapies [44,45,46,76]. Given its ability to restore adhesion, promote survival signaling, and promote resistance against IFN-γ–induced cell death and dendrite loss (Figure 6), they could be synergistically combined with JAK inhibitors, WNT agonists, or UVB phototherapy to enhance and prolong repigmentation [77,78,79]. Optimization of dosing schedules, delivery routes, and pharmacokinetics in future studies will be key to translating these findings into therapeutic strategies.

## 4. Materials and Methods

The study protocol was approved by the ethics committee of Tohoku Medical and Pharmaceutical University (2021–2–160).

### 4.1. Co-Culture Systems

Vitiligo patient-derived from primary melanocytes and human normal keratinocytes were culture as previous described [25,26]. Human iPSC-derived melanocytes [36] and human adult keratinocytes co-culture were cultured as previously described [37].

### 4.2. IFN-γ Induced Vitiligo Model

Melanocytes and adult keratinocytes were co-cultured. The ROCK inhibitor, Y27632 (10 μM) was added from day 0, and after overnight culture, fresh Y27632 only for the indicated ROCK inhibitor groups for indicated days. IFN-*γ* was added after at least three days of culture to establish stable proliferation conditions. A range of concentration refers to the reported concentrations in vitiligo patient from 0.016 to 10 ng/mL IFN-γ was added into the medium, a supraphysiological concentration compared to lesional skin levels at considerable 10–300 pg/mL, normal less than 10 pg/mL [11,12]. Media were changed daily to ensure the continued effectiveness of the ROCK inhibitor and/or IFN-γ.

### 4.3. Sampling Quality Assessment: Cell Proliferation and Relative Cell Death Analysis

The viability of iMc and adult keratinocytes was assessed using an MTS assay (CCK8, ab228554). For measuring IFN-γ induced cell death and cell proliferation percentage, cell counts were also obtained by trypan blue exclusion using a hemocytometer.

Cell Proliferation Percentage

Proliferation (%) = (N_live, treatment/N_live, control) × 100

N_live, treatment_ = mean number of viable cells in the treatment condition

N_live, control_ = mean number of viable cells in the no addition condition

Relative Death Fold Change

Relative Death Fold = N_dead, treatment_/N_dead, control_

N_dead, treatment_ = mean number of trypan blue–positive cells in the treatment condition

N_dead, control_ = mean number of trypan blue–positive cells in the no addition condition

All values represent the mean of at least three independent experiments.

### 4.4. Melanin Quantification

After co-culture, the same number of cells was calculated by trypan blue exclusion. Cells were harvested by centrifugation at 200× *g* for 5 min. The resulting cell pellet was resuspended in 100 µL of 1 N NaOH containing 10% DMSO and incubated at 80 °C for 2 h to dissolve melanin. After cooling to room temperature, the absorbance of each sample was measured at 490 nm using a microplate reader.

### 4.5. Melanocyte Dendritic Imaging Score (MDIS)

Manual Quantification

1. Dendrite Loss Rate: The percentage of melanocytes that had lost dendrites was calculated as: Loss Rate (%) = (Number of dendrite loss cells/Total number of melanocytes) × 100.
2. Dendrite Count per Cell: The number of dendrites per melanocyte was manually counted using 100× magnification images.
3. Dendritic Length: The length of each dendritic extension was measured in micrometers (μm) using the Freehand Line tool in ImageJ (NIH), based on images acquired at 400× magnification.

Sampling and Replication

For the dendrite loss rate and mean dendrite count per cell, a minimum of 100 melanocytes per condition were evaluated in three biologically independent experiments.

For mean dendritic length, all dendrites from 30 dendrite-positive melanocytes per condition were analyzed across three independent replicates, and the average value was reported.

### 4.6. RT-qPCR

RNA extraction and cDNA synthesis were performed as previously described:

Three-dimensional full-thickness skin equivalents (T-Skin^®^) were fixed and processed for histological analysis according to established protocols. Briefly, observe melanocytes. A list of primers is provided (Table S1).

### 4.7. Hematoxylin and Eosin (H&E) Staining and IF Antibodies

Following a minimum 3-day stabilization period with daily medium replacement, tissues were exposed to IFN-γ (10 ng/mL) in fresh culture medium. Paraffin-embedded specimens were sectioned at 5-μm thickness for H&E staining and immunofluorescence analysis. Antigen retrieval was performed using 0.1% Triton X-100, followed by overnight incubation with primary antibodies at 4 °C.

Sections were subsequently incubated with fluorophore-conjugated secondary antibodies (1:2000 dilution) for 30 min at room temperature and mounted using DAPI-containing medium for nuclear counterstaining.

Immunofluorescence was performed as previously described using antibodies (Table S2).

### 4.8. Fluorescence-Activated Cell Sorting (FACS)

Primary melanocytes derived from a vitiligo patient [25,26] were cultured under Rho-associated kinase (ROCK) inhibitor Y27632 conditions to enhance the efficiency and yield of human primary melanocytes in vitro. The co-culture system of these stocked melanocytes and keratinocytes were cultured in the continuous presence of Y27632 (10 μM) with or without IFN-γ (10 ng/mL) in fresh medium. Subsequently, anti-bFGF and ET-1 antibodies (Table S2) were used for FACS performance as previous report [36]. Specifically, the collected cells were fixed with 4% paraformaldehyde (PFA) for 15 min, permeabilized with 0.5% Triton X-100 for 10 min, blocked cells with the Blocking solution (1% BSA—A1595-50 mL, Sigma-Aldrich, Burlington, MA, USA) for 30 min and incubated with the primary antibody at 4 °C overnight and then incubated with the secondary antibody (647) for 30 min. After three washes, the cells were passed through a 70-μm nylon cell strainer (FALCON, 352350—Corning Inc., Tewksbury, MA, USA) and resuspended in D-PBS for flow cytometry (BD LSRFortessa X-20—BD Biosciences, Franklin Lakes, NJ, USA) according to the instrument manual.

### 4.9. Statistical Analysis

The parameters derived were expressed as mean ± standard error of the mean. Statistical method [15] used included Student’s *t*-test (two-tailed for two independent groups). ANOVA was used for comparison in more than two groups. A probability *p*-value of <0.05 was considered significant. Statistical analysis was performed using GraphPad Prism 7 (GraphPad Software, Inc., La Jolla, CA, USA).

## 5. Conclusions

In summary, our study positions ROCK inhibition at the intersection of adhesion, survival, and inflammatory signaling pathways critical for melanocyte integrity. By leveraging a tunable, patient-relevant in vitro platform, we delineated how Y-27632 restores melanocyte–keratinocyte communication and counteracts IFN-γ–mediated dysfunction. These findings lay the groundwork for combinatorial therapeutic strategies targeting Rho kinase, JAK–STAT, and WNT/β-catenin pathways—offering new hope for robust, durable repigmentation in vitiligo and related autoimmune dermatoses.

## Data Availability

MDPI Research Data Policies” at https://www.mdpi.com/ethics.

## Supplementary Materials

The following supporting information can be downloaded at: https://www.mdpi.com/article/10.3390/ijms26168069/s1.
