# Supplementary material for: Linking IFN-γ-Mediated Pathogenesis to ROCK-Targeted Therapy in a Scalable iPSCs-Based Vitiligo Model
Source: Int J Mol Sci. 2025 Aug 21;26(16):8069. doi: 10.3390/ijms26168069 (PMC12386722; doi:10.3390/ijms26168069)
Supplement: Supplementary file 1 [file ijms-26-08069-s001.zip › ijms-3805563-supplementary.pdf]

Supplementary Table 1 (Table S1):

MITF
Forward: AACCTTGCCATTGCCTGCCT
Reverse: TCTCCTGGGCTTGCTCACCT
ET1 (Endothelin 1)
Forward: CAGCAGTCTTAGGCGCTGAG
Reverse: ACTCTTTATCCATCAGGGACGAG
bFGF
Forward: ATGGCAGCCGGGAGCATCACCCACG
Reverse: TCAGCTCTTCGCAGACATTGGAAG
SCF
Forward: AAGAGGATAATGAGATAAGTATGTTGC
Reverse: TTACCAGCCAATGTACGAAAGT
Tyrosinase
Forward: CGGATCTGGTCATGGCTCCTTGG
Reverse: CACAGCAAGCTCACAAGCCC
DDR1
Forward: GGGAGAAATATAGGATAGACACTGGA
Reverse: AGTGTGTGACAGGGAGAGAGAAG
E-cadherin
Forward: AGGAGCCAGACACATTTATGGAAC
Reverse: AGTGGAAATGGCACCAGTGT
GPNMB
Forward: AAAACTGCCAGATTAACAGATATGG
Reverse: GGCAGGTCACGACAAAGTCTAT
HSD17B1
Forward: CTCGAAGGCTTATGCGAGAGTC
Reverse: GTATTGGTAGAAGCGGTGGAAG
MIF
Forward: GGACAGGGTCTACATCAACTA
Reverse: TCTTAGGCGAAGGTGGAG
GAPDH
Forward: CTCTGGTAAAGTGATATTGT
Reverse: GGTGGAATCATATTGGAACA

Table S2:

Primary Antibodies: (Table 2)

- Rabbit anti-SCF antibody (Abcam, ab64677)
- Mouse anti-ET-1 antibody (ab 2786)
- Rabbit anti-Endothelin 1 (ET-1) antibody (Abcam, ab117757)
- Mouse anti-bFGF antibody (Abcam, ab208687)
- Rabbit anti-MIF antibody (Abcam, ab7207)
- Rabbit anti-E-cadherin antibody (Abcam, ab40772)
- Rabbit anti-DDR1 antibody (Abcam, ab255810)

- Rabbit Alexa Fluor 647–conjugated anti-bFGF antibody (Abcam, ab225477)

Secondary Antibodies:

- Alexa Fluor 488–conjugated Goat anti-rabbit IgG antibody (Abcam, ab150077)
- Alexa Fluor 647–conjugated Goat anti-rabbit IgG antibody (Abcam, ab150083)
- Alexa Fluor 568–conjugated Goat anti-mouse IgG antibody (Abcam, ab150108)
- Alexa Fluor 488–conjugated Goat anti-mouse IgG antibody (Abcam, ab150113)
- Alexa Fluor 488–conjugated Donkey anti-mouse IgG antibody (Abcam, ab150107)
